# Natriuretic Peptide-Guided Therapy in Chronic Heart Failure: A Meta-Analysis of 2,686 Patients in 12 Randomized Trials

**DOI:** 10.1371/journal.pone.0058287

**Published:** 2013-03-05

**Authors:** Gianluigi Savarese, Bruno Trimarco, Santo Dellegrottaglie, Maria Prastaro, Francesco Gambardella, Giuseppe Rengo, Dario Leosco, Pasquale Perrone-Filardi

**Affiliations:** 1 Department of Advanced Biomedical Sciences, Federico II University, Naples, Italy; 2 Division of Cardiology, Ospedale Medico-Chirurgico Accreditato Villa dei Fiori, Acerra, Naples, Italy; 3 Department of Medical Translational Sciences, Federico II University, Naples, Italy; Universidad Peruana de Ciencias Aplicadas (UPC), Peru

## Abstract

**Background:**

The role of cardiac natriuretic peptides in the management of patients with chronic heart failure (HF) remains uncertain. The purpose of this study was to evaluate whether natriuretic peptide-guided therapy, compared to clinically-guided therapy, improves mortality and hospitalization rate in patients with chronic HF.

**Methodology/Principal Findings:**

MEDLINE, Cochrane, ISI Web of Science and SCOPUS databases were searched for articles reporting natriuretic peptide-guided therapy in HF until August 2012. All randomized trials reporting clinical end-points (all-cause mortality and/or HF-related hospitalization and/or all-cause hospitalization) were included. Meta-analysis was performed to assess the influence of treatment on outcomes. Sensitivity analysis was performed to test the influence of potential effect modifiers and of each trial included in meta-analysis on results. Twelve trials enrolling 2,686 participants were included. Natriuretic peptide-guided therapy (either B-type natriuretic peptide [BNP]- or N-terminal pro-B-type natriuretic peptide [NT-proBNP]-guided therapy) significantly reduced all-cause mortality (Odds Ratio [OR]:0.738; 95% Confidence Interval [CI]:0.596 to 0.913; p = 0.005) and HF-related hospitalization (OR:0.554; CI:0.399 to 0.769; p = 0.000), but not all-cause hospitalization (OR:0.803; CI:0.629 to 1.024; p = 0.077). When separately assessed, NT-proBNP-guided therapy significantly reduced all-cause mortality (OR:0.717; CI:0.563 to 0.914; p = 0.007) and HF-related hospitalization (OR:0.531; CI:0.347 to 0.811; p = 0.003), but not all-cause hospitalization (OR:0.779; CI:0.414 to 1.465; p = 0.438), whereas BNP-guided therapy did not significantly reduce all-cause mortality (OR:0.814; CI:0.518 to 1.279; p = 0.371), HF-related hospitalization (OR:0.599; CI:0.303 to 1.187; p = 0.142) or all-cause hospitalization (OR:0.726; CI:0.609 to 0.964; p = 0.077).

**Conclusions/Significance:**

Use of cardiac peptides to guide pharmacologic therapy significantly reduces mortality and HF related hospitalization in patients with chronic HF. In particular, NT-proBNP-guided therapy reduced all-cause mortality and HF-related hospitalization but not all-cause hospitalization, whereas BNP-guided therapy did not significantly reduce both mortality and morbidity.

## Introduction

Chronic Heart Failure (HF) represents a raising health care concern in developed and developing countries, reaching epidemic proportions [1]. About 1 to 2% of adult population in developed countries suffers HF, with ≥10% prevalence among elderly (>70 years) [2]. At least half of HF patients have reduced left ventricular ejection fraction, and coronary artery disease is the leading cause of chronic HF. Although in recent years progresses of pharmacologic and non-pharmacologic therapies led to substantial improvement of survival and rate of hospitalization in HF patients, prognosis remains poor [2]–[4].

Recommended pharmacological treatments in chronic HF include angiotensin-converting enzyme inhibitors or angiotensin receptor blockers, beta-adrenergic blockers, loop diuretics and aldosterone antagonists, that improve outcomes at doses used in randomized clinical trials [2]. In clinical practice, dose titration of these drugs is usually driven by assessment of patients’ clinical and volume status. However, up-titration of medications in chronic HF remains suboptimal in clinical practice, with administered doses often lower than those utilized in clinical trials, preventing achievement of the full benefit of evidence-based therapies [5], [6]. Thus, development of strategies to enhance adherence to guidelines recommended doses of drugs would be much needed to reduce the burden of mortality and morbidity in chronic HF patients.

Measurement of plasma concentrations of B-type natriuretic peptide (BNP) or N-terminal pro-B-type natriuretic peptide (NT-proBNP) is useful to rule-out diagnosis and to predict prognosis of HF patients [7]. In addition, several studies demonstrated that reduction in natriuretic peptide levels reflects the effect of therapy on cardiac loading conditions [8]–[12]. From these premises, randomized clinical trials [13]–[24] have evaluated whether adjustment of therapy to achieve pre-specified levels of natriuretic peptide levels, compared to conventional strategy mostly based on assessment of clinical status, results in more favourable mortality/morbidity in chronic HF patients. However, these trials, and two previous meta-analyses [25], [26], including some of them [13]–[20], [22], collected a small number of patients, leaving uncertain the role of this strategy in HF patient management [2], [27].

Therefore, the aim of this study was to investigate, in an updated meta-analysis including more recent clinical trials, whether a strategy of cardiac peptide-guided therapy, compared to clinically-guided therapy, favourably affects mortality and morbidity in patients with chronic HF.

## Materials and Methods

### Data Sources and Searches

This study was designed according to the PRISMA (Preferred Reporting Items for Systematic reviews and Meta-Analyses) statement, as previously reported from our group [28]–[31]. MEDLINE, Cochrane, ISI Web of Sciences and SCOPUS databases were searched for articles published in all languages until August 2012.

### Study Selection

Trials were identified by the following headings: NT-proBNP-guided, BNP-guided and randomized. As example for MEDLINE the following search was performed: (“Natriuretic Peptide, Brain” OR “NT-proBNP”) AND “guided” AND “Controlled Clinical Trials, Randomized”. Additionally, we searched reference lists of retrieved articles, bibliographies of selected trials, recent reviews and guidelines as well information from colleagues to identify additional eligible studies. Inclusion criteria for a study to be included were as follows: comparison of BNP or NT-proBNP-guided therapy versus a control group in chronic HF patients; randomized protocol; report of end-points (all-cause mortality and all-cause or HF hospitalization).

### Data Extraction and Quality Assessment

Two reviewers independently screened and selected potentially eligible trials according to the inclusion criteria. Two reviewers independently read the full-text of retained studies, which were checked to avoid inclusion of data published in duplicate. Discrepancies were resolved by discussion and consensus. Data on baseline characteristics, presence of diabetes mellitus, hypertension, aetiology of HF, NYHA (New York Heart Association) class, HF therapy and pre-specified outcomes, including all-cause mortality and all-cause or HF hospitalization. Trials’ quality was evaluated by Detsky method; studies scoring <50% were considered to be of low quality, those with a score of >75% were deemed to be of high quality, those with a score of ≥50% and <75% were designated to be of moderate quality [32](Table 1).

**Table 1:** Baseline Characteristics.

Trial	Year	Treatment(n)	Control (n)	Type of Peptide	Women (%)	Age (yrs)	Ischaemic Aetiology(%)	HTN (%)	DM (%)	NYHA class	LVEF (%)	ACE-I or ARB (%)	BB (%)	MRA (%)	Loop Diuretic(%)	Follow-up (yrs)	Detsky Quality Score
**Troughton^13^**	2000	33	36	NT-proBNP	23.2	70.1	73.9	65.2	13.0	2.0	27.0	NA	NA	NA	NA	0.79	90%
**Beck da Silva^14^**	2005	21	20	BNP	65.9	65.0	41.5	NA	NA	2.5	22.4	NA	NA	NA	NA	0.33	90%
**STARS-BNP^15^**	2007	110	110	BNP	42.3	65.5	46.8	NA	NA	2.3	30.9	99.1	98.2	23.2	100.0	1.25	85%
**TIME-CHF^16^**	2009	251	248	NT-proBNP	34.5	76.5	57.5	70.9	34.5	0.0	29.8	94.8	78.6	40.5	93.4	1.5	81%
**BATTLESCARRED^17^**	2010	121	243	NT-proBNP	36.0	75.7	59.1	43.7	17.9	2.1	38.7	NA	NA	NA	NA	3	90%
**SIGNAL-HF^18^**	2010	126	124	NT-proBNP	28.8	77.5	NA	54.8	20.0	2.4	32.0	93.6	77.6	20.0	68.4	0.75	86%
**PRIMA^19^**	2010	174	171	NT-proBNP	42.9	72.2	21.2	NA	NA	2.1	35.8	56.5	55.9	18.6	62.3	2	86%
**Anguita^20^**	2010	30	30	BNP	NA	NA	NA	NA	NA	0.0	NA	NA	NA	NA	NA	1.33	NA
**Berger^21^**	2010	92	186	NT-proBNP	35.3	71.3	69.4	72.3	45.0	0.0	NA	NA	NA	NA	NA	1	85%
**STARBRITE^22^**	2011	65	65	BNP	30.0	61.0	40.8	NA	NA	0.0	20.0	90.8	NA	67.7	93.8	0.5	81%
**UPSTEP^23^**	2011	147	132	BNP	27.2	70.9	NA	28.0	31.2	2.8	NA	100.0	93.9	57.0	89.2	1	90%
**PROTECT^24^**	2011	75	76	NT-proBNP	15.2	63.3	56.3	52.3	41.1	0.0	26.9	81.5	96.0	41.7	91.4	0.83	90%

NT-proBNP: N-terminal-pro-B-type natriuretic peptide; BNP: Brain natriuretic peptide; HTN: Hypertension; DM: Diabetes mellitus; NYHA: New York Heart Association; LVEF: Left ventricular ejection fraction; ACE-I: Angiotensin converting enzyme inhibitor; ARB: Angiotensin receptor blocker; BB: Beta-blocker; MRA: Mineralocorticoid receptor antagonist; NA: Not available. Data on NYHA class, age, follow-up and LVEF are reported as mean.

**Figure 1:**
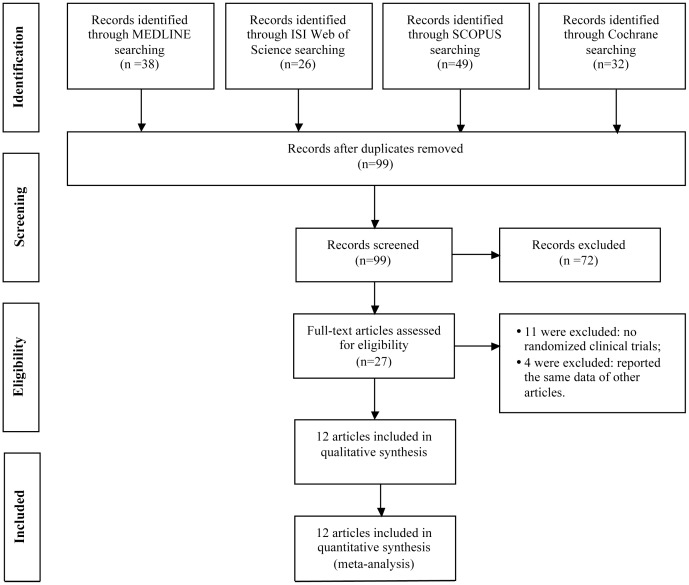
Flow chart showing the progress through the stages of the meta-analysis.

Of 145 articles identified by the initial search, 72 were excluded by title and 27 were retrieved for more detailed evaluation. Afterwards 15 studies were excluded (for instance no randomized clinical trials or reporting the same data of other articles) and the presence of the same data published in duplicate papers was resolved choosing the article reporting more information. Therefore, 12 were included in meta-analysis (Figure 1). Included trials and populations’ details are listed in Table 1. Five studies compared BNP-guided therapy to usual care [14], [15], [20], [22], [23] and 7 compared NT-proBNP-guided therapy to usual care [13], [16]–[19], [21], [24].

### Data Synthesis and Analysis

Odds ratios (OR) of the effect of randomized treatments were calculated using the metan routine (STATA Statacorp, version 11.0) [33]. OR and 95% Confidence Interval (CI) for each outcome were separately calculated for each trial, with grouped data, using the intention-to-treat principle [34]. The choice to use ORs was driven by the retrospective design of the meta-analysis, based on published studies that vary in design, subjects’ population, treatment regimen, primary outcome measure and quality [35]. Pooled ORs were logarithmically transformed and weighted for the inverse of variance. Overall estimates of effect were calculated with a fixed-effects, random effects model or Peto’s method [36] when appropriate. The assumption of homogeneity between the treatment effects in different trials was tested by Q statistic and further quantified by I^2^ statistic. A significant heterogeneity was defined by a p≤0.10 at Q statistic and by I^2^>30%, whereas I^2^<40% might indicate a not important heterogeneity. The significance level for the overall estimates of effect and for meta-regression analyses was set at p≤0.05. The first objective of the study was to separately investigate the effects of peptide-guided therapy on all-cause mortality and on HF-related hospitalization. Additionally, we investigated the effects of peptide-guided therapy on all-cause hospitalization and the effect of age on peptide-guided therapy using a composite outcome including all-cause mortality and HF-related hospitalization. Finally, we sought to assess differences between BNP- vs NT-proBNP-guided therapy on each of the above outcomes.

### Sensitivity Analysis

To explore the influence of potential effect modifiers on results, meta-regression analyses were performed with the metareg command [37](STATA Statacorp, version 11.0) to test demographic characteristics of the study population, percent of patients with DM, percent of patients with hypertension, percent of patients with ischaemic-related HF, current therapy, length of follow-up, year of publication and quality of trials [32]. For all meta-regression analyses, random effects model was used [38]. To estimate the additive (between-study) component of variance tau-2 the restricted maximum likelihood (REML) method was used to take into account the occurrence of residual heterogeneity, not explained by the potential effect modifiers [38]. To verify the consistency of the results, the influence of individual studies on the summary effect estimate (one study removed meta-analysis) was assessed using the metaninf command (STATA Statacorp, version 11.0) [39].

### Publication Bias

To evaluate potential publication bias, a weighted linear regression was used, with the natural log of the OR as the dependent variable and the inverse of the total sample size as the independent variable. This is a modified Macaskill’s test, that gives more balanced type I error rates in the tail probability areas in comparison to other publication bias tests [40].

## Results

### Characteristics of Included Trials

Baseline characteristics of 12 trials included in the meta-analysis are shown in Table 1. Of 2,686 patients, 730 were enrolled in trials comparing BNP-guided therapy to usual care and 1,956 in trials comparing NT-proBNP-guided therapy to usual care. Mean follow-up duration was 1.2±0.7 years. The overall mean age of subjects was 70±6 years and 33% were women. Characteristics of patients enrolled in BNP or in NT-proBNP trials are reported in Table 2.

**Table 2:** Baseline characteristics in NT-proBNP- and BNP-guided therapy treatment groups.

	BNP	NT-proBNP
**Treatment, No.**	373	872
**Control, No.**	357	1084
**Follow-up, mean (SD), years**	0.9±0.4	1.4±0.8
**Women, No. (%)**	235(35.1)	660(33.7)
**Age, mean (SD), years**	65.6(4.1)	72.3(4.9)
**Ischaemic Aetiology, No. (%)**	173(44.2)	904(46.2)
**Hypertension, No. (%)**	NA	975(49.8)
**Diabetes Mellitus, No. (%)**	NA	483(24.7)
**NYHA Class, mean (SD)**	2.5(0.4)	2.2(0.2)
**LVEF, mean (SD),(%)**	24(5.7)	31.7(4.7)
**ACE-Is or ARBs, No. (%)**	615(96.6)	1025(91.5)
**BBs, No. (%)**	478(96.0)	924(77.0)
**ARAs, No. (%)**	298(49.3)	379(30.2)
**Loop Diuretics, No. (%)**	591(94.4)	990(78.9)
**Detsky Quality Score**	86%	87%

NT-proBNP: N-terminal-pro-B-type natriuretic peptide; BNP: Brain natriuretic peptide; NYHA: New York Heart Association; LVEF: Left ventricular ejection; ACE-I: Angiotensin converting enzyme inhibitor; ARB: Angiotensin receptor blocker; BB: Beta-blocker; ARA: Aldosterone receptor antagonist; NA: Not available.

### Outcomes Analysis

#### All-cause mortality (Figure 2)

Natriuretic peptide-guided therapy (using either BNP- or NT-proBNP-guided) led to a significant reduction of all-cause mortality (OR: 0.738; 95% CI: 0.596 to 0.913; comparison p = 0.005; heterogeneity p = 0.896) without heterogeneity among studies. When separately analyzed, NT-proBNP-guided therapy significantly reduced all-cause mortality (OR: 0.717; 95% CI: 0.563 to 0.914; comparison p = 0.007; heterogeneity p = 0.692), whereas BNP-guided therapy did not (OR: 0.814; 95% CI: 0.518 to 1.279; comparison p = 0.371; heterogeneity p = 0.823).

**Figure 2:**
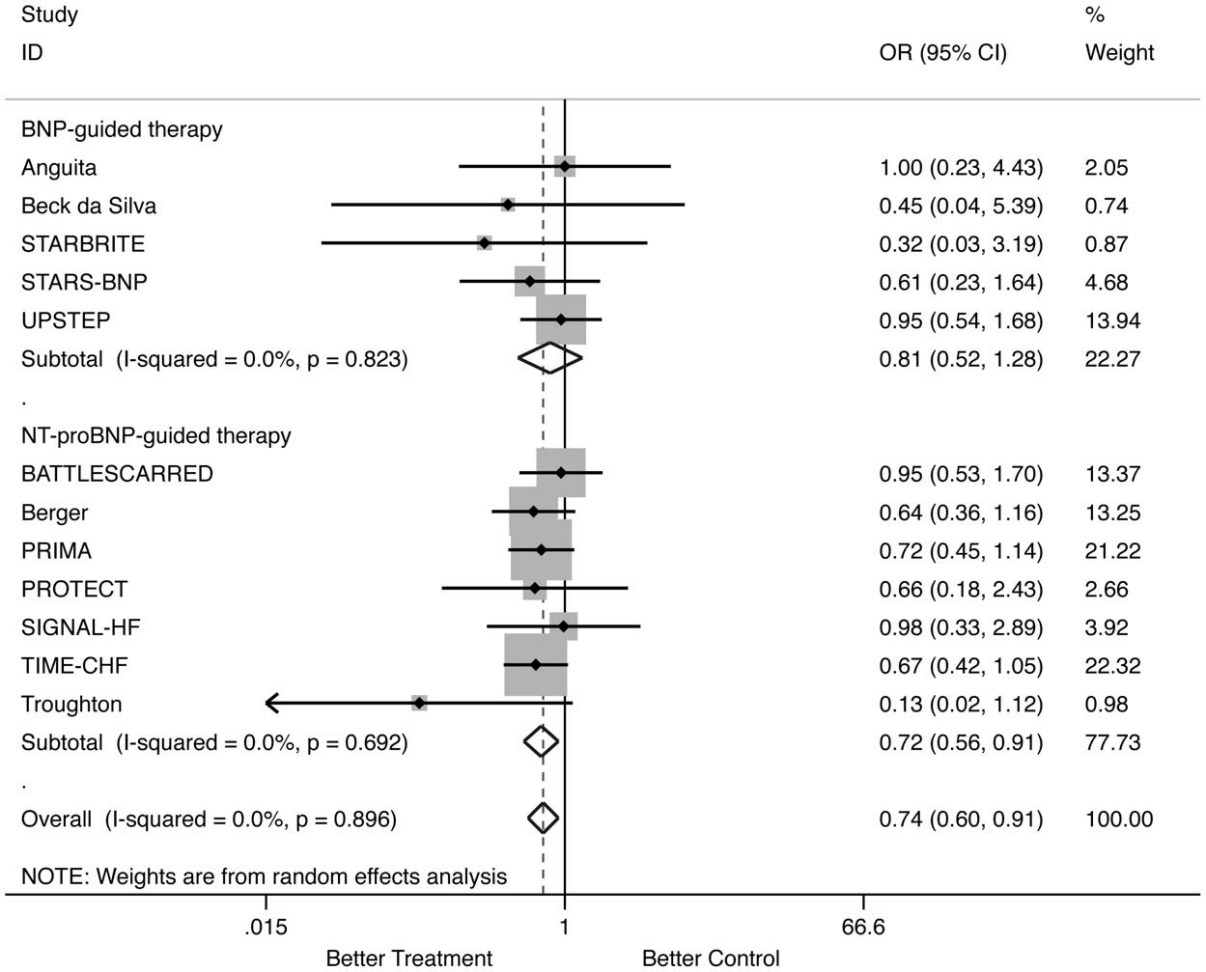
Odds ratios of all-cause mortality. Solid squares represent odds ratios in trials and have a size proportional to the number of events. The 95% confidence intervals for individual trials are denoted by lines and those for the pooled odd ratios by empty diamonds.

#### HF-related hospitalization (Figure 3)

Natriuretic peptide-guided therapy led to a significant reduction of HF-related hospitalization (OR: 0.554; 95% CI: 0.399 to 0.769; comparison p = 0.000; heterogeneity p = 0.019). When separately assessed, NT-proBNP-guided therapy significantly reduced HF-related hospitalization (OR: 0.531; 95% CI: 0.347 to 0.811; comparison p = 0.003; heterogeneity p = 0.032), whereas BNP-guided therapy did not (OR: 0.599; 95% CI: 0.303 to 1.187; comparison p = 0.142; heterogeneity p = 0.045). According to the Cochrane Handbook [41], heterogeneity among studies resulting in this analysis was resolved when 2 outlying trials [15], [17] were excluded, fully confirming the results (all trials analysis – OR: 0.546, 95% CI: 0.393 to 0.759, comparison p = 0.000, heterogeneity p = 0.151; NT-proBNP analysis – OR: 0.459, 95% CI: 0.319 to 0.661, comparison p = 0.000, heterogeneity p = 0.240; BNP analysis – OR: 0.819, 95% CI: 0.528 to 1.269, comparison p = 0.371, heterogeneity p = 0.689).

**Figure 3:**
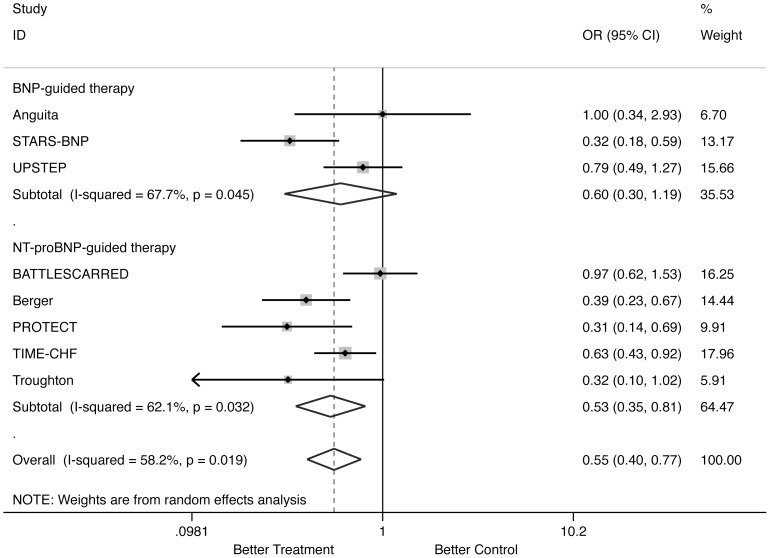
Odds ratios of heart failure-related hospitalization. Solid squares represent odds ratios in trials and have a size proportional to the number of events. The 95% confidence intervals for individual trials are denoted by lines and those for the pooled odd ratios by empty diamonds.

#### All-cause hospitalization (Figure 4)

Natriuretic peptide-guided therapy did not reduce significantly all-cause hospitalization (OR: 0.803; 95% CI: 0.629 to 1.024; comparison p = 0.077; heterogeneity p = 0.604)(Figure 3) without heterogeneity among studies. When separately assessed, neither NT-proBNP-guided therapy (OR: 0.779; 95% CI: 0.414 to 1.465; comparison p = 0.438; heterogeneity p = 0.181), nor BNP-guided therapy (OR: 0.726; 95% CI: 0.509 to 1.035; comparison p = 0.077; heterogeneity p = 0.836) significantly reduced all-cause hospitalization. For this outcome, the effect was dominated by TIME-CHF trial [16], since after its removal the reduction of all-cause hospitalization determined by natriuretic peptide-guided therapy became significant (OR: 0.689; 95% CI: 0.494 to 0.962; p = 0.029).

**Figure 4:**
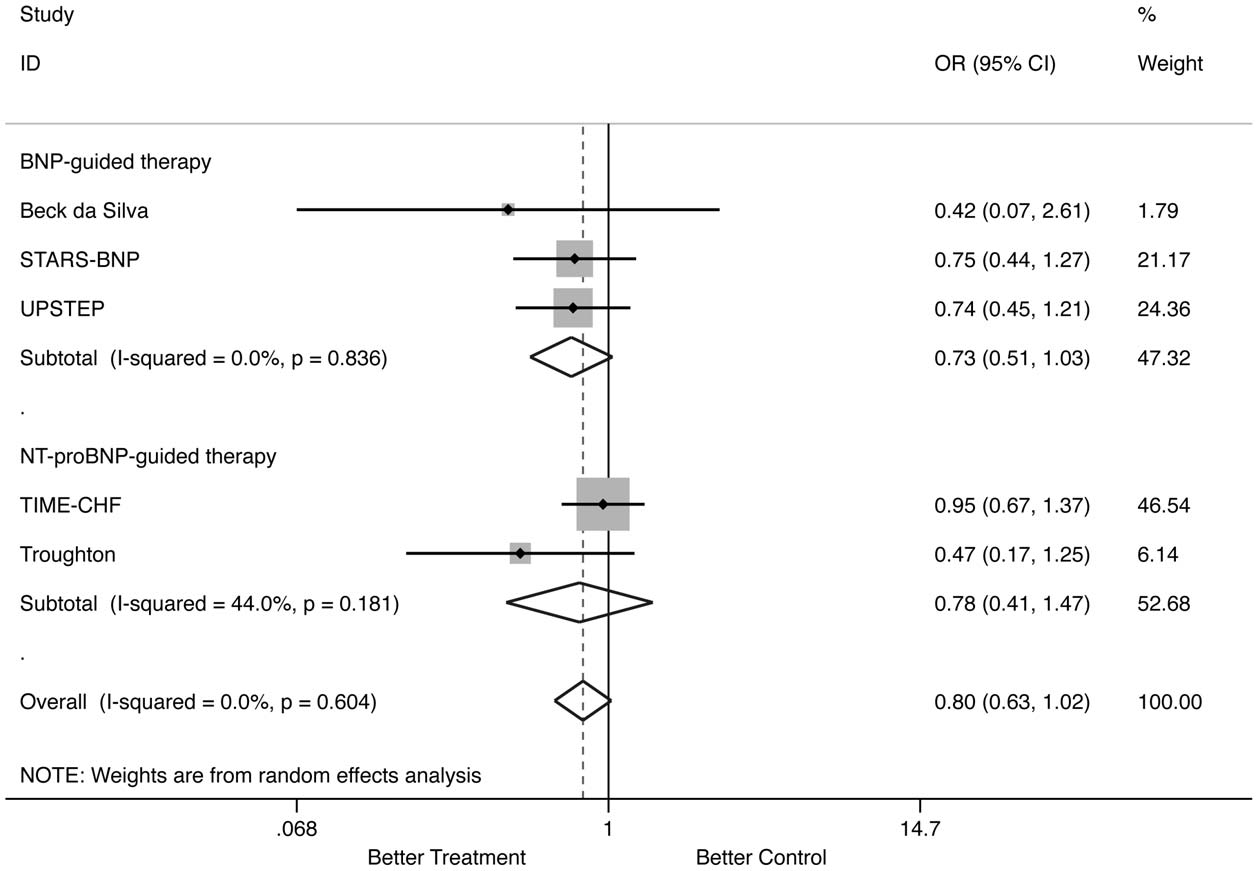
Odds ratios of all-cause hospitalization. Solid squares represent odds ratios in trials and have a size proportional to the number of events. The 95% confidence intervals for individual trials are denoted by lines and those for the pooled odd ratios by empty diamonds.

#### Younger vs older patients

Separate outcome analyses on patients younger or older than 75 years were performed using data reported in 3 trials [16], [17], [23]. The composite outcome of all-cause mortality and HF-related hospitalization was significantly reduced by natriuretic peptide-guided therapy in younger patients (≤75 years)(OR: 0.449; 95% CI: 0.207 to 0.973; p = 0.043), but not in older patients (>75 years)(OR: 0.800; 95% CI: 0.423 to 1.513; p = 0.493).

### Sensitivity Analysis

**Figure 5:**
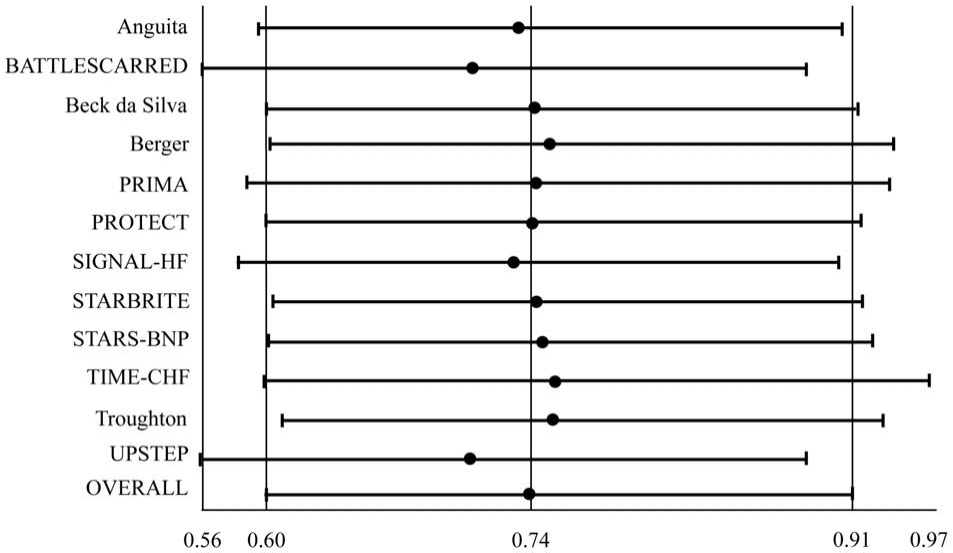
One study removed analysis for all-cause mortality. Rows represent the results of meta-analysis of all studies except the omitted study named in that row.

**Figure 6:**
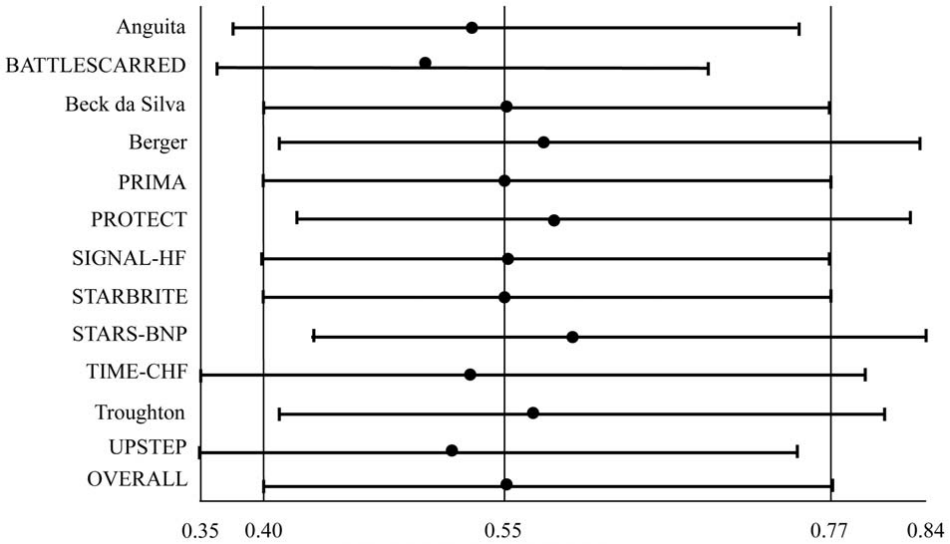
One study removed analysis for heart failure-related hospitalization. Rows represent the results of meta-analysis of all studies except the omitted study named in that row.

**Figure 7:**
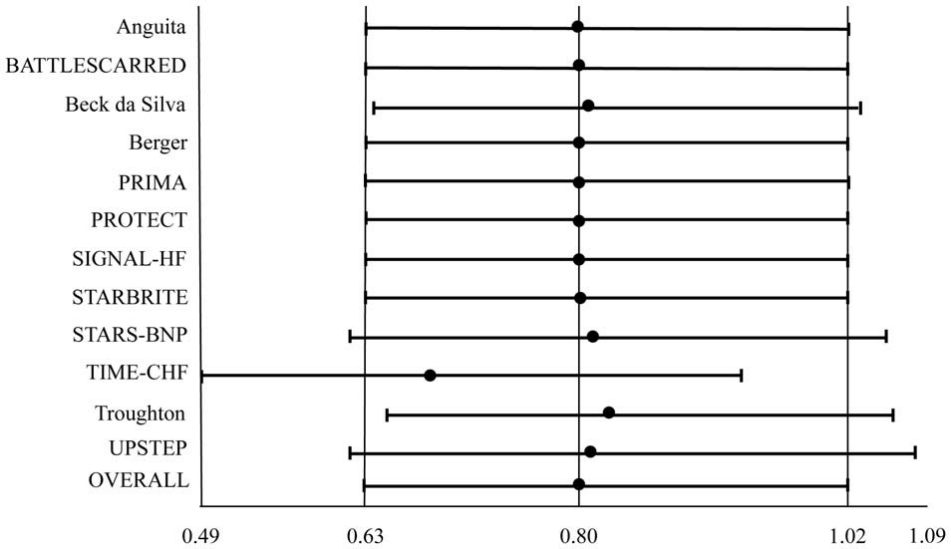
One study removed analysis for all-cause hospitalization. Rows represent the results of meta-analysis of all studies except the omitted study named in that row.

Results for each outcome were confirmed when potential effect modifiers were introduced as covariates in the meta-regression analysis (Table 3). Meta-analyses assessing the effect of natriuretic peptide-guided therapy on all-cause mortality and HF-related hospitalization were performed removing each study at a time, and in no cases removal of a single study affected the significance of the results (Figures 5,6). Additionally, removal of TIME-CHF trial [16] made significant the reduction of all-cause hospitalization determined by natriuretic peptide-guided therapy (Figure 7).

**Table 3:** Influence analysis of potential effect modifiers on the outcomes.

	All-cause mortality		HF-related hospitalization		All-cause hospitalization	
	Tau	*P* value	Tau	*P* value	Tau	*P* value
**Year of publication**	1.76	0.108	1.21	0.271	1.03	0.379
**Women**	−0.30	0.768	0.41	0.696	0.05	0.964
**Age**	0.72	0.488	2.38	0.064	1.14	0.338
**Ischaemic Aetiology**	−0.23	0.828	−0.02	0.987	−0.30	0.789
**NYHA Class**	0.68	0.525	0.43	0.709	NA	NA
**LVEF**	1.35	0.219	2.06	0.132	1.05	0.406
**Follow-up**	0.76	0.464	1.97	0.096	1.53	0.223
**ACE-Is or ARBs**	0.24	0.821	0.84	0.491	NA	NA
**BBs**	0.31	0.769	−0.85	0.487	NA	NA
**ARAs**	0.30	0.773	1.84	0.207	NA	NA
**Loop Diuretics**	−0.25	0.814	−1.33	0.314	NA	NA
**DM**	−0.73	0.498	−1.82	0.143	NA	NA
**Hypertension**	−1.37	0.230	−1.33	0.255	NA	NA
**Detsky Quality Score**	1.14	0.283	1.13	0.310	−0.90	0.435

NYHA: New York Heart Association; LVEF: Left ventricular ejection; ACE-I: Angiotensin converting enzyme inhibitor; ARB: Angiotensin receptor blocker; BB: Beta-blocker; MRA: Mineralocorticoid receptor antagonist; DM: Diabetes mellitus; NA: not available.

### Publication Bias

Macaskill’s modified test did not show publication bias for any outcome.

## Discussion

The findings of the present study indicate that, in patients with chronic HF due to systolic dysfunction, adjustment of pharmacologic therapy guided by natriuretic peptide measurements significantly reduces all-cause mortality as well HF-related hospitalization.

### Previous Studies

Natriuretic peptide levels reflect cardiac loading conditions [42], [43] and predict adverse cardiac events in patients with asymptomatic or symptomatic HF [10]. As cardiac natriuretic peptides are simply to obtain an objective marker of disease severity in HF, their role as therapeutic guidance has been investigated in several clinical trials. These studies mainly investigated two potential strategies of using cardiac peptides in the management of HF patients. The first, reported in the recent NorthStar trial [4], assessed whether high-risk but stable chronic HF patients, identified as those with NT-proBNP levels >1000 pg/mL, would benefit from prolonged specialized HF clinic assistance compared to referral back to general practitioners, and demonstrated no differences in the composite of mortality and hospitalization for cardiac causes, suggesting that the basal value of cardiac peptides has limited value to select out of hospital management strategy in HF patients. The second approach consists of targeting pharmacologic therapy on pre-specified levels of cardiac peptides, to optimize the effects of drugs. This approach has received much interest and has been tested in several trials that yielded conflicting results, with some studies demonstrating mortality or morbidity benefit from peptide-guided therapy [13], [15], [21], [24], others reporting benefit only in younger patients [16], [17] or only in responder patients [23], and other studies showing no advantages of peptide-guided compared to clinically-guided therapy [14], [18]–[20], [22]. To overcome uncertainty produced by conflicting results of single studies, 2 previous meta-analyses investigated the usefulness of natriuretic peptide-guided therapy in chronic HF [25], [26]. These meta-analyses, collecting 6 [13], [15]–[17], [19], [22] or 8 [13]–[20] randomized clinical trials, reported non definitive results on all-cause mortality and no benefit on hospitalization afforded by peptide-guided therapy, leading the recent European Society of Cardiology Guidelines on chronic HF [2] to consider still uncertain and without recommendations the use of cardiac peptides to assist management of patients, and the AHA/ACC HF Guidelines [27] to give a low-level of recommendation (IIb) to peptide-guided HF therapy, with both guidelines soliciting additional evidence.

In fact, previous meta-analyses substantially differ from the current one. In the meta-analysis by Felker et al. [25] only 6 studies were collected reporting 1,627 patients. Although a significant benefit on all-cause mortality in patients assigned to peptide-guided therapy was reported, the analysis was limited by the inclusion of 3 still unpublished studies, which prevented a detailed collection of patients’ population characteristics. Besides, the effects on all-cause or HF-related hospitalization were not analyzed. The more recent and largest meta-analysis by Porapakkham et al. [26] included 8 studies in 1,726 patients. In this analysis the favorable effect on all-cause mortality in patients assigned to peptide-guided therapy was mostly driven by the TIME-CHF trial [16], as in the sensitivity analysis the statistical significance of the effect was lost when this trial, but not any other trial included in that meta-analysis, was removed from the analysis. Notably, no difference was observed in all-cause or HF-related hospitalization. Moreover, in both previous meta-analyses [25], [26], no separate analysis for BNP- or NT-proBNP-guided therapy was performed.

Thus the current meta-analysis substantially adds to previous studies for several aspects. First, compared to previous meta-analysis, it provides evidence of benefit from larger number of studies (12 instead of 8) and of patients (2,686 vs 1,726), including more recent clinical trials in which up to date optimized pharmacologic HF therapy was used. In addition, this meta-analysis for the first time reports a clear and substantial benefit in HF-related hospitalization, which represent the main morbidity outcome in HF patients, profoundly interfering with quality of life as well with health cost expenditure. Third, the mortality benefit observed was quite consistent and not influenced, in sensitivity analysis, by any single study or by any potential confounders, which, together with the lack of significant heterogeneity, significantly strengthens the robustness of the result. Similarly to previous meta-analysis [26], we also observed no significant benefit in elderly patients, analyzing elderly subgroups from 3 trials [16], [17], [23]. Although it is conceivable that more frequent presence of comorbidities [44] may prevent or even make potentially harmful up-titration of HF drugs in elderly patients, this finding should be interpreted with caution as it comes from subgroup analysis of only 3 trials. However, use of actual rather tan age-stratified values may also have influenced the results in the elderly group [45]. Finally, at difference with previous meta-analyses, the higher number of patients included in our study allowed to separately investigate the effects of BNP- and of NT-proBNP-guided therapy, suggesting that NT-proBNP- but not BNP-guided therapy was significantly associated to improved survival as well reduced hospitalization. This finding could be explained by more favorable characteristics of NT-proBNP compared to BNP, including higher circulatory levels and longer stability, as well as reduced *in vitro* degradation [46].

### Limitations

There are limitations to this study that need to be acknowledged. First, it was not a patient-level but an aggregate data meta-analysis. Second, the findings of the separate analysis for BNP- and NT-proBNP-guided therapy need to be interpreted with caution since no single trial was designed to head-to-head compare BNP- vs NT-proBNP-guided therapy. Besides, patients enrolled in BNP trials had lower average ejection fraction compared to NT-proBNP and were better treated, possibly indicating a sicker population, with reduced room for drug up-titration. Finally patients enrolled in NT-proBNP trials were more than twofold compared to those enrolled in BNP trials, which may have prevented to observe significant association in the latters. Thus, our findings are in this regard provisional and deserve further investigation in *ad hoc* designed trials. In addition, although the findings of the study indicate that natriuretic peptide-guided therapy is associated with outcome benefits, the target peptide level to reach cannot be defined from the current findings. Finally, the possibility that our findings were influenced by more aggressive pharmacologic therapy in randomized to study compared to control arms cannot be excluded, although it does not detract the relevance of the results. However, there are also strengths of the current study. In fact, at variance with previous studies [25], [26], use of a random rather than fixed-effect model and of ORs instead of Relative Risk, made more rigorous the statistical analysis and strengthened its robustness.

### Conclusions

Use of cardiac peptides (BNP or NT-proBNP) to guide pharmacologic therapy in patients with chronic HF is associated with a significant reduction of mortality and HF-related hospitalization, especially in patients younger than 75 years. In particular, NT-proBNP-guided therapy is associated with reduced all-cause mortality and HF-related hospitalization but not all-cause hospitalization, whereas BNP-guided therapy is not significantly associated with reduced mortality and morbidity. The findings of the present study may be of help for defining the role of this approach in HF guidelines and in clinical practice.
