# Correction: Natriuretic Peptide-Guided Therapy in Chronic Heart Failure: A Meta-Analysis of 2,686 Patients in 12 Randomized Trials

**DOI:** 10.1371/journal.pone.0096706

**Published:** 2014-04-25

**Authors:** The PLOS ONE Staff

There is an error in the last sentence of the section titled “Methodology/Principal Findings” in the Abstract. The reported value of the confidence interval for all-cause hospitalization is incorrect. The correct values for all-cause hospitalization, as reported correctly in the text and figures, are (OR:0.726; CI:0.509 to 1.035; p = 0.077).
